# “Police have more standing” vs. “I don’t need the cops to stand in for me”: public perspectives on police-initiated ERPO petitions in California

**DOI:** 10.3389/fpubh.2026.1911318

**Published:** 2026-09-10

**Authors:** Nicole Kravitz-Wirtz, Alexandra Dent, Maria Cruz, Sativa Banks, Amanda J. Aubel, Shani Buggs, Veronica A. Pear

**Affiliations:** 1 Centers for Violence Prevention, Department of Emergency Medicine, University of California Davis School of Medicine, Sacramento, Davis, CA, United States; 2 Department of Public Health Sciences, University of California Davis School of Medicine, Davis, CA, United States

**Keywords:** extreme risk protection orders, firearms, health equity, law enforcement, public opinion, violence prevention

## Abstract

**Introduction:**

Extreme risk protection order (ERPO) laws, known as gun violence restraining order (GVRO) laws in California, allow temporary recovery of firearms from individuals at substantial risk of harming themselves or others. Although law enforcement officers are the most common ERPO petitioners, even in states where family members and others are authorized to petition, public preferences regarding police involvement in the petitioning process remain underexplored.

**Methods:**

We use data from the 2024 California Safety and Wellbeing Survey, a repeated cross-sectional general population online survey of California adults, to estimate the prevalence of support for and opposition to police-initiated petitions and thematically analyze open-ended responses describing the reasons for these preferences.

**Results:**

While nearly two-thirds (63.2%; *n* = 2,188) of survey respondents favored police-initiated petitions, write-in responses illuminated nuanced and context-dependent tradeoffs situated within racialized legal, political, and economic systems. These tradeoffs included: (1) systems knowledge versus relational and contextual knowledge; (2) mainstream credibility versus direct lines of communication; (3) procedural burden versus personal capacity and responsibility; (4) fear of repercussions versus wariness of law enforcement; and (5) law enforcement training and risk assessment versus alternative crisis response options.

**Discussion:**

Findings underscore the importance of ERPO implementation strategies that are responsive not only to legal and procedural requirements but also to the social and relational contexts in which decisions about petitioning occur. More broadly, findings add support to growing efforts within the field, catalyzed in part by community organizing efforts around health justice and racial equity, to assess, adapt, and scale peer- and community-based navigation approaches that complement existing implementation processes and help ensure more equitable access to the preventive benefits of ERPOs for reducing firearm-related harms.

## Introduction

**Figure 1:**
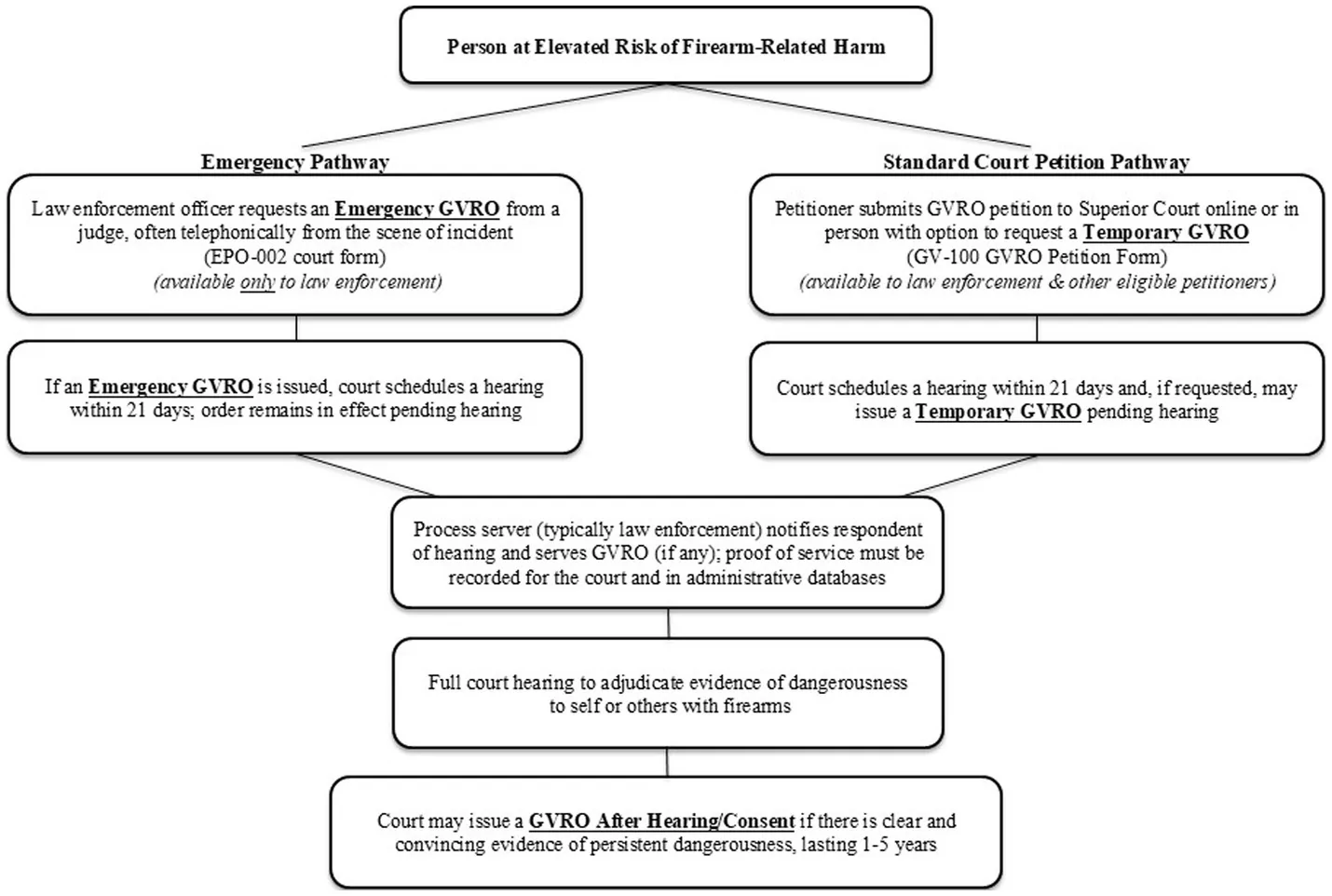
Overview of GVRO petitioning pathways in California.

Extreme risk protection order (ERPO) laws in the United States (US), also known as gun violence restraining order (GVRO) or “red flag” laws, provide a civil court mechanism to temporarily suspend the possession and purchase of firearms and ammunition by individuals judged to be at significant risk of causing harm to themselves or others. These laws are based on the well-established theoretical and empirical premise that during periods of heightened risk, temporary reductions in access to highly lethal means can decrease short-term mortality while preserving opportunities for intervention and stabilization (1). As of June 2026, ERPO laws are in effect in 22 states, the District of Columbia, and the US Virgin Islands. Although procedures vary across states, the ERPO implementation process generally proceeds in two stages (1): a judge reviews a petition filed by an eligible petitioner (typically *ex parte*), which in California may be initiated through either an emergency or standard petitioning pathway and may result in a short-term ERPO; and (2) a full court hearing is held, typically within 21 days, to evaluate the evidence and determine whether to issue a longer-term order, which may remain in effect for up to 5 years, depending on the jurisdiction (Figure 1).

Studies on the impacts of ERPO laws have produced mixed but generally promising results, particularly in cases of suicide, which account for most (>60%) firearm-related deaths (2, 3). In an analysis of ERPO use across six states, suicidal threats, plans, or ideation were identified as the precipitating event in nearly half of all ERPO petitions (4). This and other studies also found that law enforcement officers most often served as petitioners, accounting for 50 to 98% of ERPO filings, even in states where others (e.g., relatives, partners, coworkers, clinicians, educators) are also authorized to petition (4–7). Given these findings, and because overall ERPO use remains below levels likely necessary to fully and equitably optimize their benefits (6), it is important to better understand public willingness and preferences regarding the role of law enforcement officers in initiating or facilitating ERPO petitions.

Law enforcement officers operate within, and often are the most visible representatives of, criminal legal and immigration enforcement systems that disproportionately surveil, suspect, contact, arrest, and convict structurally vulnerable and minoritized people and communities (8–10). Consequently, perceptions of law enforcement officers as appropriate or preferred intermediaries within the ERPO petitioning process are likely to vary across population subgroups, especially because ERPOs are often considered in highly personal and relational contexts involving close colleagues, friends, or family members who may be in crisis or at risk of causing harm. We are aware of at least two California-based studies in which Black and Latine survey respondents reported lower levels of support for ERPOs compared with other racial and ethnic subgroups, with suggestive evidence that mistrust of police and legal institutions contributed to these differences (11, 12). Relatedly, a review of California ERPO case records found that Black and Latine individuals who were subject to ERPOs were less likely to have family or household members serve as petitioners or sources of information to petitioners, which the authors suggested may reflect concerns about engaging with the courts or involving police (11).

Public perceptions of law enforcement involvement are therefore important because they may shape whether, how, and by whom ERPO laws are used in practice. Petitions initiated by police versus other eligible petitioners differ not only in who brings the petition before the court but also in the real and perceived institutional knowledge, responsibilities, and interactions required of the petitioner. These differences may influence willingness to pursue an ERPO, experiences with the petitioning process, and ultimately the distribution of the law’s benefits and potential harms across populations. Further research is needed to enable members of the public themselves to articulate the complexity of their perceptions regarding law enforcement involvement in the ERPO implementation process, including the implications for accessibility, acceptability, behavioral health equity, and procedural justice. To better understand the factors shaping these patterns, this general population survey study from California thematically analyzes write-in responses to open-ended questions inviting respondents to explain why they would or would not prefer law enforcement officers to initiate an ERPO for a family member of concern rather than petitioning themselves. This thematic analysis builds directly on prior quantitative findings from the same survey showing that approximately one-third of California adults did not prefer police involvement in initiating an order (12). In interpreting the qualitative themes from the current study, we place particular emphasis on barriers and facilitators to petitioning and possible adjustments to the ERPO implementation process. As an early adopter of the law and a geographically, ethnically, and racially diverse state where the vast majority (~98%) of ERPO petitions are filed by law enforcement officers (4, 13, 14), we believe California’s experience can be broadly informative for equitable implementation and refinement of ERPO laws and related firearm injury prevention policies in other jurisdictions throughout the country.

## Methods

### Data source

Data are from the 2024 California Safety and Wellbeing Survey (CSaWS) Adult Questionnaire. CSaWS is a repeated cross-sectional survey designed by the Centers for Violence Prevention at the University of California, Davis and administered online in English and Spanish by the survey research firm Ipsos. Survey respondents were members of the Ipsos KnowledgePanel, an online research panel that recruits members using probability-based sampling methods with random-digit-dialing and address-based sampling from the US Postal Service’s Delivery Sequence File. KnowledgePanel members who were at least 18 years of age and non-institutionalized residents of California households were eligible to participate in CSaWS. Initial invitations were sent by e-mail from November 4–25, 2024; automatic reminders were e-mailed 3 days later and periodically thereafter. Of 5,841 invitations, 3,631 individuals completed the survey (completion rate, 62.2%); upon completion, Ipsos provided all respondents with a modest incentive and entered them into the KnowledgePanel sweepstakes (15). Analyses excluded 100 cases for refusing more than half of the survey items (*n* = 13), providing an invalid or non-California zip code (*n* = 49), or refusing to provide a zip code (*n* = 38), resulting in a final sample of 3,531 respondents. The median survey completion time was 23 min.

CSaWS was issued an exempt determination by the University of California, Davis Institutional Review Board. Therefore, formal consent was not obtained; respondents read consent language at the beginning of the survey, and progression to the next webpage constituted consent.

### Measures

CSaWS included questions about firearm ownership, exposure to violence and its consequences, and opinions on health and safety policies, including ERPO laws, which is called a GVRO law in California. Respondents were asked a series of questions regarding their awareness of the GVRO law, perceptions of its appropriateness, and willingness to initiate a GVRO for a family member of concern across five risk-based scenarios. Following this sequence, respondents were asked, “Would you prefer to have the police ask a judge for a GVRO for you?” Depending on their answer (yes/no), respondents were then asked to write-in why or why not based on the following open-ended question: “In your own words, please tell us why you would [prefer/not prefer] to have the police ask a judge for a GVRO for you?” Full-text survey items for the GVRO module are listed in Appendix 1.

### Analysis

Write-in responses to the 2 open-ended questions of interest (prefer/not prefer) were analyzed using an iterative, team-based thematic approach with a reflexive, consensus-based coding process. Responses were extracted and, as appropriate, translated from Spanish into English using a web-based translation engine; translations were reviewed for accuracy by a bilingual member of the research team (MC). After a detailed reading of the excerpts, 4 team members (NKW, AD, SB, MC) developed theory-driven and data-driven codes using an iterative process in which all team members independently coded the same sample of excerpts (*N* = 25 per question) to calibrate question-specific codebooks, refine code definitions, and develop a shared understanding of code application through regular team discussion and consensus. Once the codebooks were finalized, including 7 codes and 4 sub-codes for responses preferring police involvement and 6 codes and 7 sub-codes for responses that did not prefer police involvement, team members independently coded subsets of the remaining excerpts. Following the coding process, team members independently sorted the codes into candidate themes, which were discussed, consolidated, and finalized at team meetings. Illustrative quotes that could exemplify each theme were identified in the context of the broader narrative of the data and based on the research objectives. All excerpts were coded and analyzed in Microsoft Excel.

## Results

Of 3,531 respondents, 3,464 (98.1%) answered the closed-ended GVRO preference question, including 63.2% (*N* = 2,188) who favored having the police initiate a petition on their behalf and 36.8% (*N* = 1,276) who did not. Survey-weighted population prevalence estimates for this and other closed-ended questions from the GVRO module have been reported elsewhere (12). Among respondents who answered the closed-ended question, 1,694 (77.4%) of those who favored police involvement and 961 (75.3%) of those who did not provided valid write-in responses explaining their preferences. Themes are presented as 5 contrasts emerging from these responses (Table 1).

**Table 1:** Contrasting themes in public preferences regarding police-initiated ERPO petitions.

Prefer police-initiated petitions	Illustrative quote	Do not prefer police-initiated petitions	Illustrative quote
Systems knowledge	“They know the system and can get my request where it needs to go.”	Relational/contextual knowledge	“Family understands the situation better than any outside group and is more directly affected.”
Mainstream credibility	“Police have more ‘standing’ than a normal citizen, and would add weight to the request.”	Direct lines of communication	“I would like to have direct communication with a judge.”
Procedural burden	“I would not want to deal with the legalities.”	Personal capacity/responsibility	“If it’s my family then I should take responsibility.”
Fear of repercussions	“I would not want to risk retaliation against me or my family.”	Wariness of police	“I do not trust the police very much.”
Police training and risk assessment	“Police have the training and experience to evaluate the severity of a threat of gun violence.”	Alternative response options	“Maybe I do not want police involved in what could be a medical or mental health problem which necessitates the order.”

### Contrast 1: systems knowledge versus relational and contextual knowledge

Preferences regarding who should initiate a GVRO reflected differences in the weight respondents assigned to distinct types and sources of knowledge, which we characterize as *Systems Knowledge* and *Relational and Contextual Knowledge*. Those who preferred police involvement emphasized the advantages of institutional expertise, describing law enforcement as actors who “know the system and can get my request where it needs to go,” “know how to fill out the forms in order for the most expeditious response,” and have “better access to the court system” and a “better chance for approval.” Police were also described as more experienced and strategically positioned, as they “see this situation more often than I do and know what to say and how to say it to the judge,” with “more knowledge about the system and what to include in the request.” Efficiency was a central component, with respondents noting that officers “know how to deal with the courts [and] file the appropriate paperwork” and “más rápido obtener la [get it faster],” while also being less constrained by emotional ties.

In contrast, respondents who preferred not to rely on police underscored the importance of relational and contextual knowledge of the person of concern and their background and lived experiences, emphasizing that “en el caso de un familiar preferiría ser yo, que conozco o podría conocer todos los antecedentes de mi familiar [in the case of a family member, I would prefer it to be me, someone who knows, or could know, all of my relative’s background]” and that “family understands the situation better than any outside group.” These respondents expressed concern that police “may not know the whole story” and “tend to employ a ‘one size fits all’ approach,” whereas they themselves “could go into a lot more detail with the judge.” Some voiced distrust outright: “I do not trust the police to get it right,” while others emphasized the value of personal judgment and connection to the person of concern, noting that “my judgment is better than a policeman who may not be fully aware of the extent of the problem.” Taken together, these themes highlight an underlying tension between procedural efficiency and relational insight in decisions about GVRO initiation.

### Contrast 2: mainstream credibility versus direct lines of communication

A second dimension of respondents’ preferences contrasted *Mainstream Credibility* with *Direct Lines of Communication*, distinguishing indirect influence through institutionally conferred authority from direct, personal engagement with the GVRO process and judicial decision-makers. Those who favored police involvement emphasized externally bestowed credibility, describing police as inherently more believable actors whose words would “carry more weight” and “pack more punch with the judge.” Respondents suggested that officers “would be taken more seriously,” “may have more credibility,” and “would be able to be a more credible source that the judge would believe,” with some noting that “if police ask, I feel it would carry more weight with a judge” and that “el juez tomaria mi peticion mas seria [the judge would take my petition more seriously].” This perceived legitimacy was often linked to expectations of influence and access, with police described as having “more ‘standing’…and add[ing] weight to the request,” “more ‘pull’ in front of a judge than an ordinary citizen,” and “more power to move the judge.”

In contrast, respondents who preferred not to rely on police emphasized the importance of maintaining a direct line of communication with the judicial system, expressing a desire to “have direct communication with a judge and get all of my information firsthand” and to have their “questions answered” without intermediaries. These respondents underscored concerns about misrepresentation, stating “I do not trust the police to get it right,” and emphasized the value of speaking for themselves, with one respondent noting that “my own emotions in the situation might carry more weight.” Together, these themes reflect a tension between leveraging the institutionally conferred credibility of police and preserving direct, unmediated communication to ensure accurate and authentic representation.

### Contrast 3: procedural burden versus personal capacity and responsibility

A related contrast in preferences centered on *Procedural Burden* versus *Personal Capacity and Responsibility*, contrasting respondents who primarily emphasized the procedural burden of engaging with the legal and/or court systems with those who primarily emphasized assuming the petitioner role themselves through personal responsibility and agency. Among those who preferred the police to initiate a GVRO, perceptions of procedural burden were reflected in discomfort with navigating legal procedures and a preference for delegating the role of petitioner to someone perceived as better equipped to navigate the perceived complexities of involvement in the judicial process, with respondents noting “I’m not comfortable asking,” “I would not want to deal with the legalities,” and “I’m sure it’s complicated.” Even when willing to petition in principle, some respondents expressed uncertainty and lack of confidence in the process: “I’d be willing but uncomfortable.”

Conversely, respondents who did not favor involving the police emphasized personal capacity and ownership, framing petitioning as a responsibility that should be taken on directly, particularly in the context of family relationships: “If it’s my family then I should take responsibility” and “If I’m involved I would take the responsibility for asking for what I need.” These respondents underscored the importance of accountability and self-determination, asserting that it is often “too easy for someone else to do it for you. If you think it’s appropriate, take ownership and do it yourself” and “ONLY I CAN MAKE THAT CHOICE!!!!!” (emphasis in the original). Some stressed their own capability to act without intermediaries: “I can do it myself,” “I can act on my own if I think it necessary,” and “I am capable of speaking for myself.” Others explicitly rejected the need for police involvement: “If I’m going to do something I’m going to do it, I do not need the cops to stand in for me.” Together, these themes reflect contrasting orientations toward the petitioner role rather than mutually exclusive perspectives, with respondents differing primarily in whether they emphasized the perceived complexities and unfamiliarity of the court system or personal responsibility, autonomy, and confidence in their ability to act directly.

### Contrast 4: fear of repercussions versus wariness of law enforcement

Respondents’ preferences were also distinguished by concerns about personal, relational, and legal consequences that may be mitigated by police involvement versus concerns about the risks and trustworthiness of such involvement, which we label as *Fear of Repercussions* contrasted with *Wariness of Law Enforcement*. Among those who preferred the police to initiate a GVRO, some respondents described fear of retaliation from the subject of a GVRO and a desire for anonymity to shield themselves and their families, noting “I would not want to risk retaliation against me or my family” and “I would not want the person to know it was me asking…I would be afraid of retaliation.” Some emphasized that police could better manage potential fallout: “If something was to happen, they can address it,” while also highlighting relational concerns, such as the possibility that “the person [subject of a GVRO] will be very angry and it could disrupt relationships for years to come.” Others suggested that police involvement might redirect or diffuse personal blame. As one respondent stated, “the police asking would be more neutral and prevent unnecessary upset,” while others focused on police petitioners as a means to sidestep their own concerns related to “confidentiality” and potential “legal repercussions.” Notably, some respondents distinguished between types of risk, indicating greater willingness to act directly in cases of self-directed harm but heightened fear when interpersonal violence was a concern: “If I were afraid of someone, I would fear they would hurt me for asking for a GVRO. If I thought someone would hurt themself, I would think I would be able to ask the judge myself.”

In contrast, respondents who did not favor police involvement expressed wariness about the system of law enforcement itself, raising concerns about safety, misuse of authority, and mistrust. These respondents worried that “too many police are too quick to pull the trigger” and expressed broader skepticism, such as “I do not trust police officers to do anything involving human or animal safety” and “I do not trust the police very much.” Others questioned police discretion and potential bias, suggesting officers might be “eager to ask for one [a GVRO] even in cases where it’s not necessarily needed” or, conversely, they may be “predisposed to uphold gun rights over community safety” and “might not take the threat seriously enough.” Together, these themes reflect a tension between seeking police protection from potential interpersonal and legal repercussions and avoiding perceived risks associated with police involvement, including concerns about safety and bias.

### Contrast 5: law enforcement training and risk assessment versus alternative crisis response

A final contrast in preferences centered on *Law Enforcement Training and Risk Assessment* versus *Alternative Crisis Response*, distinguishing reliance on the perceived competencies and skills of police from a preference for alternative responders and other forms of crisis response. Among those who favored police involvement, respondents emphasized officers’ professional expertise and training, describing them as better equipped to assess risk and determine the severity of a situation. As one respondent noted, “they have more experience with situations and may be a better judge of the situation and danger than I would be,” while others highlighted that police “have far more experience in dealing with these problems” and “have the training and experience to evaluate the severity of a threat of gun violence.” This perceived competence was often linked to notions of objectivity, with respondents suggesting that police could provide “an impartial, experienced perspective” and that involving a third party reduces the likelihood of decisions driven by “a personal axe to grind.” Some also framed this role as consistent with law enforcement’s broader mandate: “That’s what they are supposed to be there for, to help and protect.”

In contrast, respondents who did not prefer police involvement emphasized that law enforcement may not be the most appropriate type of responder in these situations, particularly when crises are understood as medical and mental or behavioral health related. As one respondent explained, “this may be more appropriate for a community officer…or maybe a mental health professional,” while another noted, “I do not want police involved in what could be a medical or mental health problem.” Others framed GVRO initiation as outside the scope of police work, stating “it’s not the job of the officer,” “I believe I can self-advocate and let the police do their own job,” and emphasizing that the police are “too busy, [I] can handle it myself.” Together, these themes reflect a contrast between perceived law enforcement expertise to assess and manage risk and a preference for alternative responders based on the circumstances and needs of the person at risk.

## Discussion

Law enforcement officers are the most common ERPO petitioners, even in states such as California where permissible petitioners include not only police but also family and household members, intimate partners, employers, coworkers, school employees, and teachers. This general population survey study of California adults provides qualitative insights into public perceptions of ERPO implementation, particularly the petitioning process and the role of law enforcement as de facto intermediaries within it. While approximately two-thirds of respondents reported preferring police to initiate an ERPO petition on their behalf, thematic analysis of open-ended survey responses suggests that preferences regarding police involvement reflect nuanced and sometimes competing considerations, revealing a series of context-dependent tradeoffs situated within larger legal, political, and economic systems that shape people’s lives and wellbeing. The patterns we observe highlight tensions and opportunities for refinement across key domains related to the ERPO implementation process, including knowledge and experience; authority, legitimacy, and representation; efficacy, responsibility, and engagement; protection and harm; and response appropriateness and competence.

Prior survey research in California and nationally has documented widespread public support (>75%) for ERPOs as an appropriate violence prevention tool, both in general and in scenarios in which survey respondents would consider initiating an order for a family member experiencing cognitive decline, emotional distress, or threatening to harm themselves or others (12, 16). Yet high levels of public support, including self-reported willingness to personally serve as a petitioner, have not translated into comparable levels of ERPO use for real people during periods of elevated risk. Our findings suggest that this gap is not solely a function of awareness or attitudes toward the law itself—though, public awareness of ERPO laws remains relatively low in California and nationally (2, 12)—nor simply a matter of potential petitioners choosing between involving police and acting independently. Rather, it represents a broader set of barriers and facilitators that may not be fully addressed within the existing ERPO implementation landscape.

While some survey respondents viewed police as possessing systems knowledge, implicit credibility, and bureaucratic efficiency, these presumed advantages were counterbalanced by others’ concerns about safety, bias, and misalignment between law enforcement involvement and the nature of the crisis, concerns that may be reinforced by prior evidence suggesting insufficient training and misperceptions about ERPO laws among police officers themselves (17). Conversely, while family members and other civilian petitioners may possess deep relational and contextual knowledge about the person of concern, they may lack the self-perceived expertise, confidence, or willingness to get involved, including knowledge of firearm-related safety planning (18), and may be reluctant to engage directly with legal and enforcement systems for various ideological and experiential reasons related to procedural justice (19). Taken together, these findings add support to growing interest across the field, catalyzed in part by larger community organizing efforts around health justice and racial equity, in assessing, adapting, and scaling peer navigation approaches (20, 21) to augment existing implementation processes and address unmet needs in ERPO utilization (22).

Such alternative facilitators could include trained community-based organization staff, community violence interventionists, unarmed crisis (co-)responders, legal and court advocates, or other non-law enforcement actors with specialized ERPO knowledge and community credibility who are equipped to support individuals through the potentially burdensome petitioning process. These roles are implicitly reflected in survey respondents’ accounts that emphasize the need for guidance (“I wouldn’t want to deal with the legalities”), concern about misrepresentation (“I don’t trust the police to get it right”), and a preference for alternatives to law enforcement involvement (“I would prefer not to have law enforcement nervous or uncertain around the person”), particularly in situations understood as medical or behavioral health related.

These civilian- or community-based liaisons could help translate procedural requirements into broadly accessible language, assist with documentation and court preparation, provide emotional support, and facilitate communication between petitioners and the court, thereby addressing barriers identified in evaluations of similar laws in other states, where the majority of petitions filed by non-law-enforcement petitioners (depending on the order type, 61 to 65%) were rejected because of procedural errors or deficiencies rather than insufficient evidence of dangerousness (23, 24). In doing so, they could build on facilitators identified in our findings, such as credibility, efficiency, and person-centered care, including the ability to coordinate resources and referrals grounded in mental health, social service, and harm reduction rather than law enforcement, while simultaneously addressing barriers related to institutional knowledge, self-efficacy, representation, and mistrust of formal systems without sacrificing the relational and contextual knowledge that respondents viewed as critical to effective decision making.

This approach aligns with the proactive, non-punitive spirit of ERPO laws, as well as broader calls to invest in community-centered systems of safety and alternative response models that support compassionate care, risk assessment, and social service delivery, while also allowing law enforcement to streamline their focus on serious crimes in progress. In such cases, as well as during ERPO service and firearm recovery, law enforcement may remain a preferred or necessary actor. However, ensuring that potential petitioners have access to multiple entry points, including coordinated peer- and community-based supports, may enhance both the reach and equity of ERPO use, particularly among populations that have expressed lower levels of trust in legal and law enforcement systems and who may therefore be less likely to benefit from the preventive impacts of ERPOs on both other-directed, and especially, self-directed harm. This is particularly important given evidence that recent increases in suicide rates have been disproportionately steep among Indigenous and Black individuals in the US, alongside research showing that exposure to community gun violence, which itself is unequally distributed across communities, is a significant risk factor for suicide (25).

### Limitations

Our findings should be considered in light of several limitations. First, self-reported data is subject to nonresponse and social desirability biases; however, item nonresponse for closed-ended questions was low overall, and research suggests that completion rates are higher and social desirability bias is lower in online panel surveys than in random-digit-dial telephone surveys (26). Self-reported preferences also may not reflect real-world behavior in situations involving acute risk, where decisions about ERPO petitioning are shaped by time constraints and emotional stress. Responses to open-ended questions nonetheless provided key insights into respondents’ perspectives on real-world barriers and facilitators, even though many were brief and may not have captured the full complexity of decision making. Third, while the sample was drawn using probability-based methods, it may not fully represent individuals most directly affected by ERPO use or firearm violence and suicide, including those with lived experience as petitioners or those subject to ERPOs. Additionally, although we think these findings can inform understandings of ERPO implementation and refinement in other states, variations in firearm ownership, the cultural acceptability of firearms, firearm violence, and firearm regulations should be considered when attempting to generalize these results beyond California. Fourth, the study focuses on perceptions of the petitioning process and does not address preferences at later stages of the ERPO process (e.g., service of the order and firearm recovery). Finally, thematic analysis is inherently interpretive, and while conducted rigorously, alternative interpretations and less common perspectives may not be fully reflected.

## Conclusion

Findings from this statewide general population survey study underscore the importance of designing ERPO implementation strategies that are responsive not only to legal and procedural requirements but also to the social and relational contexts in which decisions about petitioning occur. Efforts to increase ERPO uptake should therefore extend beyond awareness campaigns to include investments in navigation support and clear pathways to crisis responses that are appropriate to the nature of the situation, as well as partnerships with community-based organizations engaged in violence prevention and alternative behavioral health response. By aligning implementation with the diverse orientations toward knowledge, authority, responsibility, risk, and competencies identified in this study, such efforts may enhance the accessibility, acceptability, and equity of ERPOs as a tool for preventing firearm-related harms.

## Data Availability

The datasets presented in this article are not readily available because analyses by the primary research team are still ongoing, after which data from the 2024 California Safety and Wellbeing Survey will be made publicly available. Requests to access the datasets should be directed to UC Davis Centers for Violence Prevention, cvp@health.ucdavis.edu.
